# Autoimmune liver diseases in Latin America: Current landscape and challenges

**DOI:** 10.1097/HC9.0000000000000830

**Published:** 2025-12-12

**Authors:** Cláudia Alves Couto, Alejandra Villamil, Debora Raquel Benedita Terrabuio, Sofia Tejada, Graciela Castro-Narro, Ezequiel Ridruejo

**Affiliations:** 1 Hospital das Clínicas da Universidade Federal de Minas Gerais, Instituto Alfa de Gastroenterologia, Belo Horizonte, Minas Gerais, Brazil; 2 Hospital Italiano de Buenos Aires, Buenos Aires, Argentina; 3 Hospital das Clínicas da Universidade de São Paulo, São Paulo, Brazil; 4 Gastroenterology, Hepatology and Liver Transplant Department, National Institute of Medical Sciences and Nutrition “Salvador Zubirán,” Mexico City, Mexico; 5 Hepatology and Liver Transplant Unit, Hospital Medica Sur, Mexico City, Mexico; 6 Hepatology Section, Department of Medicine, Centro de Educación Médica e Investigaciones Clínicas Norberto Quirno “CEMIC,” Buenos Aires, Argentina

**Keywords:** autoimmune hepatitis, epidemiology, health disparities, health services accessibility, liver transplantation, primary biliary cholangitis, treatment outcome

## Abstract

**Figure:**
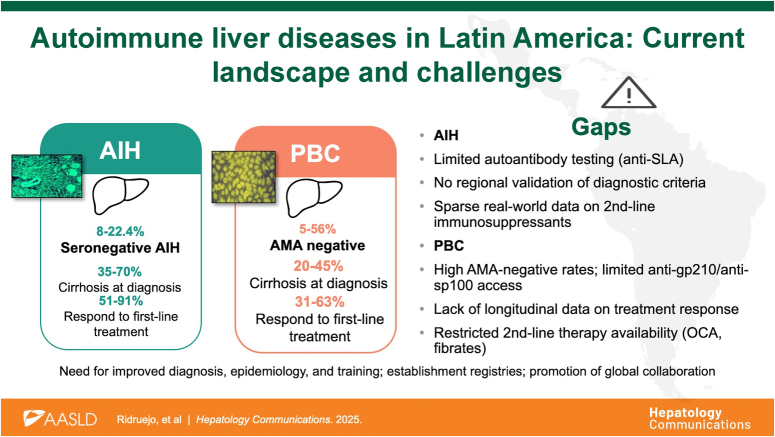

## INTRODUCTION

Autoimmune liver diseases (AILD), primarily autoimmune hepatitis (AIH) and primary biliary cholangitis (PBC), are uncommon but clinically significant causes of chronic liver disease worldwide. They are characterized by immune-mediated injury to hepatocytes or cholangiocytes, leading to progressive inflammation, fibrosis, and, ultimately, liver failure or liver transplantation. Despite emerging data regarding the global incidence and prevalence of AILDs,^1^^,^^2^ robust epidemiological data from Latin America remain scarce. Nevertheless, regional studies suggest that these diseases represent a non-negligible proportion of the liver disease burden, accounting for up to 22% of liver transplant indications in selected centers in Brazil, Argentina, and Mexico.^3^^–^^5^ The ACLARA study on acute-on-chronic liver failure in Latin America suggests that as many as 10% of hospitalized patients had an underlying AILD diagnosis.^6^ Data from the United States also underscores the severity of AILD in Latin America. Recent U.S. studies show that Hispanic patients with AIH tend to have more severe disease, Hispanics have the highest rate of cirrhosis diagnosis (55%),^7^ and are hospitalized for AIH at a rate 20% higher than Whites.^8^ For all these reasons, the burden of AILDs in Latin America deserves close inspection. Herein, we review the current knowledge on AILD in Latin America, detailing the epidemiological, clinical, immunological, and therapeutic particularities, as well as the key gaps in care and research that persist within the region—and the tools needed to address them.

### The unique needs of Latin Americans with AILD

Latin America is a wide geographical region composed of 20 countries and inhabited by 645 million people. It is characterized by a great ethnic diversity as a result of varying proportions of Amerindian, Asian, African, and European genetic ancestries, shaped by local interactions with migrants brought by the slave trade, European settlements, and local indigenous populations.^9^ This particular racial and ethnic composition has a direct impact on the expression of disease and predisposes to wide regional variations, as a result of significant genetic heterogeneity.^9^^,^^10^ In addition, the region is challenged by inadequate access to healthcare. Public systems are underfunded, overburdened, and unevenly distributed. Barriers in access for patients to timely diagnosis and treatment, particularly for high-cost medications, foster significant inequalities.^11^^–^^13^ Liver transplantation is not widely available and is further hindered by low organ donation rates. Access is also constrained by structural and geographic inequalities, including vast territorial dimensions, uneven distribution of transplant centers, and disparities in health policy and infrastructure among countries. These barriers disproportionately affect patients living outside major urban centers and contribute to delays in referral and treatment. Moreover, access to subspecialists in hepatology and the availability of immunosuppressive therapies also remain challenging. These gaps result in serious deficits for both our understanding of AILD epidemiology and our ability to address the needs of patients with AILD in Latin America.^14^^,^^15^

## AUTOIMMUNE HEPATITIS IN LATIN AMERICA

### Epidemiology and ethnic disparities

AIH is a chronic and progressive immune-mediated liver disease that affects all ages, both sexes, and all ethnicities, with a marked female predominance in both adults (71%–95%) and children (60%–76%).^10^ Hallmarks of AIH include hypergammaglobulinemia, autoantibodies, interface hepatitis on histology, and response to immunosuppressive therapy. According to American Association for the Study of Liver Diseases (AASLD) guidelines, AIH should be suspected in patients with elevated liver enzymes, positive autoimmune serologies, and after exclusion of other causes of liver disease. Its incidence and prevalence have shown an upward trend globally, with a pooled incidence of 1.28 cases per 100,000 person-years and a prevalence of 15.65 per 100,000.^10^

Significant ethnic and geographic variations in disease presentation and outcomes have been reported. Studies show that Sub-Saharan African, Indian, and Brazilian patients tend to be younger and present with more aggressive disease compared with Western populations.^1^ A recent U.S. study comparing Hispanic and non-Hispanic patients with AIH found that Hispanic individuals had higher body mass index (BMI), more frequent metabolic dysfunction–associated steatotic liver disease (MASLD), and elevated Fibrosis-4 Index (FIB-4) and Aspartate Aminotransferase to Platelet Ratio Index (APRI) scores at 1 year. They also showed delayed biochemical remission and required longer corticosteroid therapy, despite similar baseline disease severity and clinical outcomes. These findings are consistent with previous reports suggesting that AIH may present more severely in Latin American populations. While the presence of comorbidities such as MASLD is more frequent in this group, current evidence does not establish a causal relationship. It remains possible that such comorbidities contribute to delayed remission or more advanced disease at diagnosis, but other genetic, environmental, or socioeconomic factors may also play a role.^16^

In Latin America, although population-based studies are lacking, AIH appears to be a leading cause of acute liver failure requiring liver transplantation. In Argentina, it accounts for 25%–30% of transplants due to fulminant hepatic failure.^3^^–^^5^ In Peru, AIH represents 2%–4% of all transplant indications.^17^ A multicenter Mexican cohort found autoimmune liver diseases present in 17% of cirrhotic patients, reaching 25% among females.^2^

### Genetics of autoimmune hepatitis in Latin America

Although AIH pathogenesis remains incompletely understood, both genetic predisposition and environmental triggers play key roles. Among genetic factors, particular attention has been given to the human leukocyte antigen (HLA) system, which appears to significantly influence susceptibility and disease expression.^18^ Differently from Europe and North America, where type 1 AIH (AIH-1) susceptibility is linked to DRB1∗0301 and DRB1∗0401, and type 2 AIH (AIH-2) is linked to the DRB1∗0301 allele, studies in Latin America have reported distinct associations. In the 1990s, studies with adult and pediatric patients from Argentina and Brazil with AIH-1 who tested positive for anti-smooth muscle antibody (ASMA) were more likely to harbor HLA alleles DRB1∗13:01 and DRB1∗03. In Argentina, the HLA-DRB1∗03 allele was more frequent among childhood-onset cases.^19^ In Brazil AIH-2, was linked to DRB1∗07 and DRB1∗03 alleles.^20^^,^^21^

A systematic review of 694 AIH patients from Brazil, Argentina, Mexico, and Venezuela confirmed that HLA-DQ2 and DR52 groups were associated with disease risk, whereas HLA-DR3 (important in other regions) was not significant.^18^ The DRB1∗1301 allele remained a notable risk factor (OR 2.48), confirming previous findings from Brazil and Argentina, also in Mexico and Venezuela. Interestingly, this allele was also found in North Americans without DRB1∗03 and DRB1∗04 (13% of the cohort), in which 54% carried HLA-DR13. Patients with HLA-DR13 were younger at presentation when compared with patients with HLA-DR4, failed corticosteroid therapy more often than HLA-DR4 patients, and exhibited a lower relapse rate (50% vs. 85%) and higher frequency of sustained remission after treatment withdrawal (50% vs. 15%) when compared with patients with HLA-DR3.^18^

HLA profiles may influence disease presentation and progression in Latin America, as described in a comparison between 115 Brazilian and 161 U.S. AIH patients. Brazilian patients were younger, had higher AST and gamma globulin levels, lower HLA-DR4 frequency, and higher HLA-DR13 frequency.^21^

### Diagnosis of AIH in Latin America

We summarize the diagnostic data underpinning the diagnosis of AIH from Latin American tertiary care centers 2008–2025 in Table 1.^11^^,^^22^^–^^30^

**TABLE 1:** Summary of AIH studies in Latin America

Reference; country; sample size; FUT (months/year)	Age at diagnosis (y);Serological markers EHAID (%)	Clinical presentation at disease onset; Cirrhosis (%)	Treatment response	Clinical outcomes
Dávalos, M (2004);^25^ Peru; n=30 FUT NA	48.59 y ANA=73.3%; SMA=43.3%; AMA=16.7% EHAID NA	63.3% abnormal LFT and/or clinical signs of CLD 6.7% ALF F4=70% (varices=20%)	26/30—IS 80% initial response 2/26—partial 3/26 (11.5%)—non-response	16.7% complications with IS—3 severe infections Death=10% (2/3 infection)
Terrabuio, D (2008);^23^ Brazil; n=268; 6.2 y	29.1±17.1 y^a^ ANA=13.4% ANA+SMA=46.2% SMA=40.5% Anti-LKM-1=7.1% AMA=5.6% (no criteria for overlapping) Seronegative=9% EHAID=28.4%	Acute hepatitis-like=56% CLD + PH=25% Asymptomatic abnormal LFT=10% ALF=3.4% F4 in liver biopsy=56%^b^ Cirrhosis=62.3%^c^ F3/4=57.8%	BR=51.5% HR=36.2% Median time to first HR=4.04 y Mean doses of IS at HR=AZA 84.3+PD 8.3 mg/d Side effects=57.5%	HCC=4/268 5-year survival=91.4% Death=15.7% (main etiology infection) LT=9.7 Recurrence of AIH=26.9%
Landeira, G (2012);^26^ Argentina; n=139; 57.7 mo	45.7 y (13–59) AIH-1 only EHAID=27%	Acute hepatitis-like=49% CLD=35% Cirrhosis complications=16% Cirrhosis=45.8% (older, lower PA and albumin) Non-cirrhotic=higher BT, AST, ALT	Complete =91% Partial=4% Non-response =5% Similar treatment response in cirrhotic and non-cirrhotic (82% vs. 94.7%)	Cirrhotic patients— Lower Probability of remaining free of cirrhosis complications and lower 10 y survival (67.1% vs. 94.4%) Liver TX=5.7% Death=12.9% Liver-related death=55.5%
Soares, JC (2016);^24^ Brazil; n=36; 01/2013– 07/2015	46.5 y ANA=58.3% SMA=55.6% Anti-LKM-1=5.6% Seronegative=8% EHAID NA	Signs of liver failure=19.2% F3/4=77.4% Cirrhosis=35.5% Patients with significant fibrosis (N=23)—Lower AST, ALT, TB, PA	6-month liver enzymes normalization was higher in patients without significant fibrosis, lower PA, and liver failure at diagnosis	NA
Díaz-Ramírez, G (2018);^36^ Colombia; n=278; 41 mo	50 y ANA=79.1% SMA=32.4% AMA=12.2% Seronegative=16.2% EHAID=28.4%	Acute hepatitis-like=29.6% Asymptomatic /abnormal LFT=15% Inespecific symptoms + abnormal LFT=22.9% ALF=2.8% F4 in biopsy=40.1% Cirrhosis=37.8%^c^	Analysis in 245 patients. BR=84.9% Time for BR=13.4 wk Partial=12.2% Non-response=2.9% Relapse=15.9% (IS withdrawal=55.5%/ discontinuation or DR by doctor=22.2%)	20.6% developed cirrhosis during FUT Death=5.7% (MOF secondary to liver failure and related infectious complications) LT=10.1% Recurrence after liver tx=14.3%
Fedrizzi, RS (2020);^28^ Brazil; n=40; 2.7 y	44.2 y ANA=20% ANA+SMA=42.5% SMA=17.5% Anti-LKM-1=5% Seronegative=15% EHAID=20%	Acute onset=35% Compensated/ decompensated Cirrhosis=25%/12.5% Asymptomatic abnormal LFT=27.5% Cirrhosis=35%	Complete/partial=85.7% Non-response=14.3% HR=37.5%	Fibrosis regression=18.75% Fibrosis progression=18.7% HCC=0 Death=0 LT=10%
Fernández, MIC (2020);^22^ Cuba; n=82; 84 mo	46.5 y AIH-1=89% AIH-2=11% EHAID=37.8%	Asymptomatic=12.2% Acute disease=15.8% Mild or subclinical=72% Cirrhosis=39%	Remission=79% in 11.7 mo Incomplete=16% Treatment failure=4.9% IS withdrawal in 8 (BR+HR)— Relapse =75% Side effects=92.7%	Progression to cirrhosis=34.1% Progression to decompensated cirrhosis=23.2% HCC=2.4% 5-y survival=98.4% 18-y survival=44.8% Death=7.3% LT=20.7%
Cervera, V (2021);^30^ Uruguay; n=33	Only naive patients with TB >2.5 mg/dL 52 y ANA=39.4% SMA=39.4% Anti-LKM-1=3% Anti-LC1=6.1%	Cirrhosis=57% Ascites=45.5% HE=30% Median INR=1.72 MELD=24 Liver biopsy in 57.6%–42.3% F4, 26% submassive/massive necrosis, 47.4% centrilobular necrosis	IS treatment=82%/Initial liver TX=18% Complete=66.7% Partial=14.8% Non-response=18% Factors related to non-response: pretreatment MELD (non-responders=27 vs. responders=19), hypercolesterolemia and HE at baseline	5 y-overall survival=90% 5 y transplant-free survival=62.5% 5 y event-free survival: MELD<25=85% MELD ≥25=41% In CS treated patients, 5y event-free survival— MELD <25=90%, MELD ≥25=60%
Guedes, LR (2024);^27^ Brazil, n=102; 115 mo	24.5 y AIH-1=85.3% AIH-2=5.9% Seronegative=8.8% ANA=66% SMA=57% Anti-LKM-1=7%	Cirrhosis=59% (more frequent in older patients) Stage of cirrhosis 0–1=43%/2 (CSPH)=14.6%/3–5 (decompensated)=43% MELD=13 Ascites=18% 🡪 higher AST/ALT ratio and MELD score	One-year BR=55.7% HR=30.3% Cirrhosis associated with lower odds of remission Complete remission in 24—IS withdrawal in 16—relapse in 9 in 2.5 mo (56.5%)	Overall survival=89% Mean transplant-free survival=304±13 mo 10 y Survival 🡪 BR (+)=99.8% vs BR (−)=87.2% Death=4.1% LT=5.9% Ascites at disease onset=20-fold increase in mortality
Barbero, M (2025);^38^ Argentina, n=125; 01/2013– 12/2021	36.4 y ANA=68% SMA=46.4 Anti-LKM-1=2.4% Seronegative=22.4%	Patients evaluated for LT for AIH decompensated cirrhosis (125/1321, 9.5%) Two groups: IS-treated (72)—57.6% 🡪 Mean MELD=16.5/MS-Ascites=52.8%/SBP=16.7% Non-IS (53)—42.4% 🡪 Mean MELD=19.4/MS-Ascites=79.2%/SBP=18.9% Median PD dose 8 mg AZA dose was 50 mg for all Mycophenolate dose 1000 mg/day	IS improves survival 🡪 Median transplant-free survival in the IS Group=22.6 mo vs. 6.57 mo in non-IS group (*p*=0.002) Factors associated with LT or death— moderate/severe ascites and MELD-Na Analysis of patients with MELD >22 (21.6%) 🡪 loss of IS protective effect. MS-ascites increased the risk of death/LT Infection 🡪 IS group=34.7% vs. non-IS group=34.6% Most common infections in IS group (n=32)=pneumonia, UTI, bacteremia, SBP. Death in 8/25 (32%)	

^a^ Expressed in mean±SD.

^b^ Diagnosis according to liver biopsy, which was performed in 80.6% of the cohort (121/216).

^c^ Diagnosis according to clinical, radiological, endoscopic, and histological findings.

Abbreviations: AIH, autoimmune hepatitis; ALF, acute liver failure; ALT, alanine aminotransferase; AMA, anti-mitochondrial antibody; ANA, anti-nuclear antibodies; AST, aspartate aminotransferase; AZA, azathioprine; BR, biochemical remission; CLD, chronic liver disease; CS, corticosteroids; CSPH, clinical significant portal hypertension; DR, dose reduction; EHAID, extrahepatic autoimmune diseases; FUT, follow-up time; HCC, hepatocellular carcinoma; HE, hepatic encephalopathy; HR, histological remission; IS, immunosuppression; LFT, liver function tests; LT, Liver transplantation; MOF, multiple organ failure; MS-Ascites, moderate/severe ascites; NA, not available; PA, prothrombin activity; PD, prednisone; PH, portal hypertension; SBP, spontaneous bacterial peritonitis; SMA, anti-smooth muscle antibody; TB, total bilirubin.

Of note, there was substantial heterogeneity in sample size (35–278 patients), follow-up duration (41 mo to 6.2 y), and inclusion criteria across studies. The definition of cirrhosis varied, ranging from histological confirmation alone to the inclusion of clinical, radiological, and endoscopic findings. Consistent with international cohorts, a female predominance was observed (female-to-male ratio 3:1 to 9:1). Extrahepatic autoimmune diseases—particularly hypothyroidism, rheumatoid arthritis, and psoriasis—were reported in 20%–28.4% of patients, in line with global estimates (14%–44%).

Most patients had AIH-1, and AIH-2 accounted for 5%–7.1% of cases, typically with childhood onset. Seronegative AIH, defined by the absence of ANA, ASMA, and anti-LKM-1 (Liver Kidney Microsomal), was observed in 8%–15% of patients-like global reports of up to 20%. However, in North America and Germany^26^ nearly one-third of initially cryptogenic cases were later reclassified as AIH based on anti-SLA/LP (soluble liver antigen/liver pancreas) positivity, which is unfortunately not routinely available in LA. In some Latin American cohorts, anti-LKM and anti-SLA/LP were not assessed.

Acute hepatitis-like onset was the most frequent AIH presentation in Latin America (29.6%–59.4%), notably higher than international cohorts (10%–26%). Acute liver failure occurred in 2.8%–3.4%, and 7.8%–31.3% were asymptomatic. Decompensated cirrhosis was present in 6.3%–22%, more than typically reported in Europe and North America.^27^ These differences likely reflect heterogeneous definitions and delayed diagnosis. Genetic and environmental factors may also contribute.

Three studies explored severe forms of AIH presentation^30^^–^^33^. In Uruguay, 33 treatment-naïve patients with AIH and a MELD score >24 were studied. Despite acute presentation, 58% were cirrhotic, and 15% had massive necrosis at initial biopsy.^30^ In severe cases, including patients with high MELD scores, corticosteroid therapy achieved remission in two-thirds, with MELD scores <25 predicting favorable outcomes.

In Brazil, 72 patients with acute AIH were evaluated. Genuine acute AIH (GAAIH), defined by specific biochemical and histological criteria, accounted for 8.3% of cases and showed better liver function and treatment response compared with acute exacerbations of chronic AIH (67.7% vs. 15.2%).^32^

In an Argentine cohort of 86 patients with AIH-related acute liver failure (28% of all causes), nearly 80% required liver transplantation or died within 7.8 days despite treatment with methylprednisolone. Poor outcomes were associated with severe coagulopathy, grade IV encephalopathy at the initiation of corticosteroid therapy, reactivity of liver cytosol and LKM-1 antibodies, massive necrosis, and lack of early response to CS.^33^

In Latin American cohorts, cirrhosis at the time of diagnosis—defined by liver biopsy and/or clinical, radiological, and endoscopic findings—was present in 35–59% of patients, and 39.2–77.4% had advanced fibrosis (F3/F4) on initial histology.^11^^–^^13^^,^^15^^–^^20^ The proportion of cirrhosis at diagnosis was exceptionally high in a Peruvian study of 30 patients, reaching 70%, but only 20% had esophageal varices at endoscopy.^17^ These rates exceed those in European and North American studies (20%–30%) and may reflect delayed diagnosis and genetic factors. Although biopsy studies have confirmed a higher prevalence of MASLD and its association with AIH in Latino patients, these histologic findings have not been clearly linked to worse outcomes ^16^.

The simplified diagnostic criteria for AIH, previously validated in European, Asian, and North American populations, have also been assessed in Latin America. These studies are important because they confirm the diagnostic accuracy of the tool in populations with distinct genetic and clinical backgrounds. A Mexican study found higher specificity, but lower sensitivity compared with the revised criteria, while a Chilean study confirmed excellent overall accuracy, with an area under the curve of 0.98.^34^^,^^35^

### Treatment response and clinical outcomes

Limited data on initial treatment regimens and heterogeneous response criteria hinder comparisons across Latin American studies and with international cohorts. While biochemical response was typically defined by normalization of transaminases and IgG, assessment time or timing of assessment, and histological remission was often not evaluated. Despite these limitations, some cohorts have offered valuable insights.

In an Argentine cohort of 139 AIH patients (55 with cirrhosis at diagnosis) followed for 57.7 months, who received azathioprine (AZA) and CS, 91.4% achieved complete remission. Treatment failure occurred in 5%. Cirrhotic patients had similar treatment response than non-cirrhotics, but lower probability of remaining free of cirrhosis complications and lower 10-year survival.^26^

In a Colombian cohort of 278 AIH patients (37.8% with cirrhosis), 85% achieved biochemical remission with standard treatment, 85% achieved biochemical remission after a median of 13.4 weeks. Corticosteroids were withdrawn in 21.3% and all immunosuppression in 3.4%.^36^

Two Brazilian cohorts provided further insights. In one (268 patients, 56% with cirrhosis), biochemical and histological remission were achieved in 51.5% and 36.2%, respectively. In another (102 patients, 59% cirrhotic, 43% decompensated), remission rates were 55.7% (biochemical) and 30.3% (histological) at 1 year. Despite modest remission rates, outcomes—such as cirrhosis progression (18.7%–20.6%), liver transplantation (5.9%–10%), and mortality (4.1%–15.7%)—were comparable to international data.^23^^,^^27^ Couto et al.^37^ assessed autoantibodies during treatment as predictors of complete response in 117 AIH-1 patients (follow-up: 35 mo). Complete response occurred in 68%, but persistence of anti-ASMA (>1:80) and anti-actin (>1:40) was significantly linked to ongoing biochemical (76.9–79.8%) and histological (100%) disease activity.

Cirrhosis at diagnosis showed inconsistent associations with treatment response. While Colombian^36^ and Argentine studies reported lower remission in cirrhotic patients^26^, one Brazilian cohort found no effect. Acute presentation, higher albumin, and prothrombin activity predicted higher levels of histological remission.^23^ In contrast, ascites and cirrhosis were linked to poorer survival, whereas remission was associated with improved outcomes.^27^

In a study of 125 patients with decompensated cirrhosis, immunosuppression improved transplant-free survival. However, its benefit was lost in those with MELD >22, and moderate to severe ascites was associated with increased risk of death or transplantation, with infection being the leading cause of mortality.^38^

A 2024 ALEH-led multicenter study (preliminary data) included 200 patients across 7 Latin American countries.^39^ AIH-1 accounted for 93.7%. Cirrhosis was present in 39% at diagnosis. First-line therapy involved prednisone (86%), AZA (81%), and mycophenolate (8%). Biochemical remission at 12 months occurred in 66.9%. Mycophenolate was the most common second-line agent (60%), after intolerance or non-response to azathioprine. Death occurred in 10.5%, liver transplantation in 1.5%, and HCC in 1.1%.

### Challenges and future perspectives on AIH in Latin America

In 2023, a web-based survey conducted by ALEH gathered responses from 65 hepatologists across Latin America.^12^ AIH was identified as the most frequent AILD and revealed key barriers to optimal care. Anti-SLA/LP testing was largely unavailable, despite its prognostic relevance. Although liver biopsy was broadly accessible (97%), only 72.3% were assisted by liver pathologists.

Treatment indications varied, with many physicians still treating based on signs of clinical disease activity rather than elevated enzymes or IgG. Corticosteroid monotherapy was preferred in decompensated cirrhosis, and histological remission was inconsistently assessed (48% performed liver biopsy to define complete response).

Among emerging therapies, antimalarials have shown promise. These drugs are currently used for the treatment of systemic lupus erythematosus, being associated with sustained beneficial effect on overall and disease-free survival, reduction in severity of exacerbations, antithrombotic effect, improvement of metabolic profile, protection against kidney damage, and occurrence of serious infections. Antimalarials have been evaluated to treat AIH by the University of São Paulo group for the maintenance of disease remission after IS withdrawal and initial treatment.^40^^–^^42^ In 2009, chloroquine use reduced relapse rates post-IS withdrawal compared with historical controls, with 6.49 times less chance of relapse in the study group (72.2% vs. 23.5%). A randomized trial (n=61) showed relapse rates of 40.7% (chloroquine diphosphate 250 mg/d) versus 80.1% (placebo), with anti-SLA/LP positivity as a risk factor. Another study compared antimalarial + CS with AZA+CS for initial treatment in 57 patients, with biochemical response in 53.8% (antimalarial) versus 67.7% (AZA). Antimalarial was associated with improved metabolic profiles. Despite promising data, its use in AIH remains limited to a single center and warrants validation in larger studies. Their immunomodulatory, non-immunosuppressive nature offers potential benefits in selected patients.

## PRIMARY BILIARY CHOLANGITIS IN LATIN AMERICA

### Epidemiology and ethnic disparities

PBC is a chronic, immune-mediated cholestatic liver disease characterized by the progressive destruction of small intrahepatic bile ducts, leading to fibrosis, cirrhosis, and eventually liver failure. It predominantly affects women aged 40–60.^43^ There is regional variability and underreporting, especially in Latin America, which may hinder a clear epidemiological understanding.^44^^,^^45^

In recent years, some authors have reported an increase in the global prevalence and incidence of PBC. This trend is likely attributable to greater diagnostic awareness and the availability of more sensitive detection tools, including anti-mitochondrial antibodies (AMA) and specific anti-nuclear antibodies such as anti-gp210 and anti-sp100.^2^^,^^43^ Although previously described as a disease affecting primarily white non-Hispanic women, recent data have highlighted an increasing prevalence among men and underrepresented ethnic groups.^45^ In the United States, Black and Hispanic patients with PBC experience significantly higher waitlist mortality and delisting due to disease progression compared with White patients.^46^ In Canada, Indigenous patients with PBC are more commonly diagnosed at advanced stages and have lower transplant-free survival rates.^47^

In Latin America, many patients are diagnosed at advanced stages, suggesting delays in disease recognition.^48^ National registries are scarce, and limited involvement in international research networks further complicates efforts to fully understand the regional burden of PBC. There are also specific challenges related to timely access to diagnosis, therapy with ursodeoxycholic acid (UDCA), and adequate follow-up that may negatively impact the prognosis of Latin American patients. In Brazil, for example, only in 2018, UDCA was incorporated into the treatment of primary biliary cholangitis in the Public Health System. Although objective data are lacking, clinical experience indicates that patients still face challenges related to the irregular distribution of the medication, which may negatively impact adherence to treatment and therapeutic outcomes.^48^ Data on second-line therapy are scarce and refer mainly to fibrate use.^49^

Several reports from the United States have alerted over significant differences in clinical presentation of PBC, response to treatment, and prognosis between Caucasians and Hispanics living in the United States, mainly from Mexico, Central and Latin Caribbean. In a multicenter study that included 535 patients, Peters et al.^46^ observed that progression to advanced liver disease, pruritus, ascites, hepatic encephalopathy, and variceal bleeding were more frequent in non-Caucasian than in African American and Hispanic patients. Similar findings were reported by Levy et al.,^50^ who, in addition, observed in a cross-sectional study that Hispanic patients with PBC have reduced response rates to UDCA and a higher frequency of associated AIH. It has even been described as an increased waitlist mortality and a lower rate for liver transplantation for Hispanic patients with PBC. Rabbiee et al. sought to identify differences in demographics, comorbidities, environmental risk factors, and socioeconomic status between Hispanic and non-Hispanic patients with PBC to justify these differences.^51^ No significant differences were observed between the 2 groups with respect to predisposing factors, such as hair dye and nail polish use, alcohol and tobacco use, age of menarche or menopause, number of miscarriages or pregnancies, or itching during pregnancy. Yet, important socioeconomic disparities were identified, including lower levels of education, lower income, and decreased availability of health care insurance, which could play a major role in disease progression and adequacy of medical management. These socioeconomic discrepancies and their impact on access to care may further explain these variations, potentially leading to late identification of PBC, more severe disease, and lack of response to available therapies for Latin American patients with PBC living in the United States.^52^^–^^57^

Data from 482 patients with PBC living in Brazil evaluated by the Brazilian Cholestasis Study Group (28 hepatology centers) described a long time to diagnosis, a remarkable frequency of advanced PBC at diagnosis, a high incidence of pruritus (48%) and a much higher female-to-male ratio compared with other series (21:1). Limited access to UDCA, inconsistent follow-up, and restricted availability of second-line therapies were identified as major challenges. These findings highlight barriers to optimal care but limit the generalizability and comparability of treatment outcomes across the region.^48^

Initial results from Argentina,^52^^,^^53^ Chile,^54^ Uruguay^55^ and Mexico^56^ have shown similar findings in clinical features at diagnosis. Generally, a higher proportion of patients presented with advanced disease stages, suggesting delayed diagnosis.

A series from Chile among 115 patients evaluated between 1990 and 2002 has shown that a significant proportion of patients were already cirrhotic at diagnosis, with clear evidence of portal hypertension and symptomatic disease represented 78% of patients.^54^ Data from Uruguay analyzing 81 patients provides similar information describing over 78% of patients with symptoms, mainly pruritus, and nearly 40% cirrhotic at diagnosis.^55^

Though this situation might be improving in the last decade, according to results observed in Argentina by Bori et al.^52^

Table 2 summarizes the main results of PBC studies in Latin America.

**TABLE 2:** Summary of PBC studies in Latin America

Reference; country; sample size; FUT (months/years)	Age at diagnosis (y); Serological markers; sex	Clinical presentation at disease onset; Cirrhosis (%)	Autoimmune disease associated	Treatment response Clinical outcomes
Cançado GGL et al., 2022;^48^ Brazil; n=562 (+ subsets); 6.2±5.3 y	56±5 (overall), AMA+ 59.6, AMA− 52.2; ANA 66%, SMA 4.4%, IgM (↓ in AMA−); 95% female	Pruritus 48%, Fatigue 38.4%, Cirrhosis 32%; AMA-negative had earlier symptoms and slower ALP/GGT response	Hashimoto 13.8%–19.8%, Sjögren 7.8%–11.1%, RA 3.7%–8.9%, scleroderma 2.5%–6.5%	UDCA: 40.5%–63.3% 1 y. Fibrates 39%–76% (12–24 mo); deep response 6.6% LT, 3.2% liver-related deaths
Bori MS et al., 2024;^53^ Argentina; n=596; 40 y (retrospective)	57.2±12.2 (recent); 54.3±11.6 (past); ALP: 2.69–5.49 × ULN; TB: 0.92–3.45 mg/dL; AST/ALT stable; Female predominance	Symptomatic ↓ from 73.7% to 50%; advanced histology ↓ from 60.2% to 20.8%	Not reported	Not reported; Early biochemical stage ↑ from 18.0% to 77.4%
González-Huezo MS et al., 2019; Mexico; n=78; 2005–2012 (retrospective)^56^	55.8; AMA 94.8%, ANA 70.5%, SMA 8%; Female predominance	Not specified; all met PBC diagnostic criteria	62.8% with autoimmune diseases (Sjogren 29.5%, thyroid 26.9%, Raynaud 14.1%)	Not reported; Not reported
Domínguez Cardoso PF et al.,^57^ 2022; Mexico; n=60; 2015–2022 (retrospective)	Fifth–sixth decade; AMA 95%, ANA (among 26%), overlap AIH 25%; 95% female	Significant fibrosis (68%), steatosis (30%)	33% with autoimmune disease (Sjögren, scleroderma); overlap AIH 25%	UDCA response (Paris II): 31%; high fibrosis=worse outcome
Pimentel C et al., 2024;^58^ Venezuela; n=19; 2022–2024 (retrospective)	53.5±15.2; AMA 31.6%, AMA-M2 68.4%, SP-100 52.6%, GP-210 42.1%; 94.7% female	Pruritus 73.7%, Fibrosis 57.9% by FibroScan, portal hypertension 15.8%	Not reported	GP-210 is associated with higher fibrosis, bilirubin, and portal hypertension (worse prognosis); SP-100 is not predictive
Valera JM et al., 2006;^54^ Chile; n=115; 13 years retrospective	52 (range 30–76); AMA 56%, ANA 35%, AML 28%, IgM↑ 71%; 96% female	Pruritus 69%, fatigue 62%, asymptomatic 22%; Cirrhosis/fibrosis 61% at baseline	Sjögren 38%, hypothyroidism 13%, scleroderma 7%, RA 5%, Raynaud 4%	UDCA in 94%; liver transplant in 15 patients (13%); 66.7% survival at 36 months post-LT
González-Huezo MS et al., 2019; Mexico; n=78; 2005–2012 (retrospective)^56^	55.8; AMA 94.8%, ANA 70.5%, SMA 8%; 96% female	Not specified; all met PBC criteria; 73% high globulin	62.8% with autoimmune diseases: Sjögren 29.5%, thyroid 26.9%, Raynaud 14.1%, CREST 11.4%, RA 7.7%, vitiligo 6.4%, others	Not reported
Guatibonza-García V et al., 2021; Colombia; n=43; 2009–2019 (retrospective)^57^	58.7±11.4; AMA 44.2%, ANA 74.4%, ASMA 14%, IgM 57.1%; 100% female	Pruritus 46.5%, Fibrosis in 39.5% (stages II–IV)	Sjögren 20.9%, Hashimoto 14%, scleroderma 9.3%, RA 9.3%	UDCA 86%, IgM associated with fibrosis (OR 11.0); low performance of AMA as a diagnostic marker

Expressed in mean±SD.

Abbreviations: ALP, alkaline phosphatase; AMA, anti-mitochondrial antibody; ANA, anti-nuclear antibodies; FUT, follow-up time; LT, Liver transplantation; NA, not available; PBC, primary cholangitis biliary; RA, rheumatoid arthritis; SMA, anti-smooth muscle antibody; TB, total bilirubin; UDCA, ursodeoxycholic acid.

### Diagnostic methods and the role of liver biopsy

Guatibonza-Garcia et al. have alerted about the poor performance of AMA for the diagnosis of PBC among female patients in Colombia. They have identified positivity for AMA in only 44% of patients among 85 patients, requiring a liver biopsy (47%–3%) to define the diagnosis. ANA positivity was reported in 74% of patients, though they have presented no data on the presence of anti-GP210 and anti-SP100 in that group of patients.^58^

Similar results, with lower-than-expected AMA positivity, were observed in Brazil (83%), Chile (56%–65%), and Venezuela (68%).^48^^,^^49^^,^^59^ Only 15.7% of patients who tested negative for AMA-M2 showed positivity for the specific antibodies GP-210 and/or SP-100 in Venezuela. The lower AMA positivity rates reported in Latin American cohorts may indicate a greater need for liver biopsy to establish a definitive diagnosis of PBC in this population, which, in turn, may further limit access to timely diagnosis in resource-constrained settings.

Even though data from the Brazilian Cholestasis Consortium, evaluating 464 subjects (83%) AMA-positive, demonstrated that AMA-negative PBC was associated with younger age, earlier symptoms, lower IgM/TG, higher total bilirubin, but similar response treatment rates. The authors concluded that AMA-positive and AMA-negative PBC patients share similar clinical, histological, and biochemical features with AMA-positive PBC, supporting the notion that both belong to the same disease spectrum and should not be managed differently.^60^

### Management and treatment

Although UDCA is available across Latin America, there are no objective data to quantify limitations in its distribution. Nonetheless, clinical experience indicates that its real-world accessibility is limited by structural and socioeconomic barriers. Variability in healthcare system access and irregular public coverage—often dependent on government health services—frequently leads to treatment gaps and generates significant inequities within the same country. This situation disproportionately impacts individuals of lower socioeconomic status, often resulting in reduced adherence to treatment. In some cases, patients may intentionally lower the prescribed dose or interrupt treatment altogether to reduce personal expenses, thereby compromising therapeutic efficacy and long-term disease control. Most robust PBC data in Latin America comes from the Brazilian Consortium on Cholestasis, evaluating a cohort of 297 treated PBC patients. The 1-year treatment response to UDCA as first-line therapy for PBC was found to be significantly lower than expected: only 39% responded, considering Paris II criteria (ALP ≤1.5×ULN, AST ≤1.5×ULN, normal total bilirubin) and 61% for Toronto criteria (ALP ≤1.67×ULN).^48^ Similar results were observed by the Zubiran Hospital in Mexico, with response rates ranging from 20% to 47%, depending on the criteria used.^61^

The Brazilian Cholestasis group further assessed “deep response,” defined as normal ALP and bilirubin, after 1 year of UDCA treatment. Among 297 patients, only 22.9% reached deep response. Cirrhosis and elevated baseline ALP levels were associated with reduced odds of deep response. Interestingly, within a Brazilian cohort of 206 patients with PBC, incomplete response rates to UDCA were not statistically different when assessed either at 6 or 12 months using Toronto, Rotterdam, or Paris 2 criteria.^62^ Those differences were even smaller or absent in those subjects with advanced PBC. This data favors early addition of second-line therapies, especially in patients with advanced disease or high baseline liver enzyme levels in Latin America. The same group proposed a novel predictive model, ALP-A score, based on age and ALP levels at diagnosis, which proved effective in identifying patients unlikely to respond adequately to UDCA monotherapy, thus enabling earlier treatment intensification strategies.^63^

Regarding second-line treatment, only off-label use of fibrates is available in the region. Fibrates are available in all countries for treating hypertriglyceridemia. Their use is widely implemented, though the availability of different fibrates varies between countries, where bezafibrate is not available, fenofibrate or ciprofibrate are used instead.

A long-term prospective study has been carried out in Argentina by Sorda et al.,^64^ evaluating response to therapy with UDCA plus bezafibrate 400 mg/day in 31 patients with incomplete response to UDCA. Biochemical response was achieved in 86% of patients, and a significant improvement of histological parameters was observed at 5 years on follow-up biopsies, with regression of fibrosis attained in 48% of patients. Efficacy and safety of fibrates—specifically ciprofibrate and bezafibrate—as add-on therapy were also evaluated in the Brazilian cohort. Both bezafibrate and ciprofibrate were associated with significant improvement in cholestatic markers, with ~60% of patients achieving a biochemical response after 12 months of combination therapy. Ciprofibrate showed comparable efficacy to bezafibrate, and both agents were well tolerated, with no severe adverse events reported. These findings support the role of fibrates, including ciprofibrate, as effective and safe second-line options in UDCA-unresponsive PBC.^49^

A preliminary report from the ALLATIN cohort, a multicenter study across Latin America sponsored by ALEH, 231 patients with PBC from countries including Brazil, Argentina, Chile, and others were analyzed—mostly women (92%) with a mean age of 50.5 years. Cirrhosis was present in 26%, and 16% had AIH overlap. AMA was positive in 71%, and 67% achieved biochemical response to UDCA. Second-line fibrates were used in 20% of cases, mainly bezafibrate. Liver-related mortality occurred in 3%, and 6% underwent liver transplantation.^65^

Regarding PBC, the web-based survey conducted by ALEH in 2023, answered by 65 hepatologists, showed restricted access to antibodies for the diagnosis of PBC as well as AIH. Access to AMA antibodies was reported by 100%, followed by anti-smooth muscle (98.5%) and anti-nuclear antibodies (95.4%), while access to anti-gp210 and anti-sp100 antibodies was limited (55%). UDCA was the primary medication for PBC (100%), followed by fibrates. There was considerable heterogeneity in selecting the criteria to evaluate PBC response to UDCA, with 39.3% favoring Paris II criteria, yet 70% evaluated response at 6 months, particularly in high-risk patients. Although non-response or intolerance to UDCA were the principal indications for fibrates, 23% never considered adding fibrates to optimize response.^12^

### Challenges in the region and regional initiatives in PBC

Historically, PBC has received less research attention when compared with other autoimmune liver diseases. This limited focus has resulted in a slower accumulation of knowledge regarding its pathogenesis, diagnostic approaches, and optimal treatment strategies. However, in recent years, there has been a significant shift, with an increasing number of collaborative initiatives focused on advancing research and improving clinical management of PBC. These initiatives often took the form of multicenter registries, which allow for more comprehensive data collection and enable in-depth analysis and discussions tailored to specific local populations.

The strengthening of collaborative networks within Latin America is crucial for the advancement of knowledge, medical education, and access to effective treatments. Latin American countries, while facing distinct regional challenges, stand to benefit significantly from shared resources and expertise. By working together, the region can address gaps in understanding the disease and improve the overall healthcare infrastructure for its management.

Table 3 summarizes the challenges requiring urgent action in Latin America, while Tables 4, 5 highlight areas that would benefit from collaborative research in the region.

**TABLE 3:** Unmet needs and proposed research directions in autoimmune liver diseases in Latin America

Clinical/scientific area	Data gaps	Rationale for future research
Epidemiology	Incidence and prevalence estimates across Latin American countries using standardized methodologies	Critical for quantifying disease burden and informing national and regional public health planning
Diagnosis	Time to diagnosis and diagnostic access; availability of autoimmune serology and liver biopsy	Essential to understand diagnostic delays and improve early identification and staging
Seronegative phenotypes	Frequency and clinical implications of seronegative AIH and AMA-negative PBC	May influence diagnostic algorithms and the use of extended serologic or histologic evaluation
Clinical outcomes and quality of life	Symptom burden, functional impact, and patient-reported outcomes	Needed to inform therapeutic goals and improve patient-centered care
Treatment	Regulatory approval, availability, and clinical use of second-line therapies (eg, obeticholic acid, mycophenolate, fibrates)	Informs national access policies and optimal therapeutic sequencing
Treatment response	Longitudinal data on biochemical, histologic, and clinical response	Necessary to define appropriate treatment targets and evaluate real-world effectiveness
Liver transplantation	Transplant eligibility criteria, access barriers, and long-term post-transplant outcomes	Critical to reduce disparities and optimize referral pathways

Abbreviations: AIH, autoimmune hepatitis; AMA, anti-mitochondrial antibodies; PBC, primary biliary cholangitis.

**TABLE 4:** Disease-specific gaps in AIH and PBC

AILD	Domain	Identified gap	Suggested future research
AIH	Diagnosis	Lack of regional validation of simplified diagnostic criteria, especially in seronegative cases	Multicenter studies evaluating diagnostic performance and the incorporation of non-standard autoantibodies
AIH	Treatment	Limited data on real-world use and clinical outcomes of second-line immunosuppressants	Prospective observational studies on efficacy, safety, and optimal sequencing of alternative therapies
AIH	Prognosis	Insufficient data on recurrence and risk stratification post-liver transplantation	Development of national transplant databases to assess predictors of post-transplant outcomes
PBC	Diagnosis	High proportion of AMA-negative cases; limited access to anti-gp210 and anti-sp100 testing; unclear role of biopsy	Standardization of diagnostic pathways and evaluation of seronegative variants using histology and advanced serology
PBC	Treatment response	Inconsistent use of response criteria; lack of longitudinal monitoring data	Longitudinal cohort studies using harmonized response definitions to assess UDCA effectiveness
PBC	Access to second-line therapy	Limited availability and unclear outcomes of fibrate use; OCA is not widely accessible	Health policy research assessing regulatory approval, cost-effectiveness, and safety of second-line agents

Abbreviations: AILD, autoimmune liver disease; AIH, autoimmune hepatitis; AMA, anti-mitochondrial antibodies; OCA, obeticholic acid; PBC, primary biliary cholangitis;UDCA, ursodeoxycholic acid.

**TABLE 5:** Key strategies for improving the management of AILD in Latin America

Key strategies	Description
Strengthening healthcare infrastructure	Enable timely diagnosis and ensure long-term follow-up for patients with AILDs. This addresses diagnostic delays and improves access in both primary and secondary care settings.
Expanding medication availability	Improve the availability and affordability of essential medications, including immunosuppressive agents.
Healthcare professional training	Enhance education and training programs to improve recognition and management of AILDs. Ongoing education is essential to reduce clinical inertia and improve disease awareness among providers.
Public policies for transplant access	Implement policies to ensure equitable access to liver transplantation for advanced disease patients.
Development of national and multicenter registries	Create and maintain centralized databases to improve disease surveillance, inform healthcare planning, and facilitate research.
Integration of socioeconomic and ethnic variables into clinical and research frameworks	Encourage inclusion of social determinants of health in clinical assessments and research protocols to reduce disparities.
Fostering collaborative and international research	Support multicenter research initiatives to generate robust data reflecting the region’s diversity. Addresses underreporting and facilitates integration into international networks. Facilitate inclusion of Latin American centers in international consortia to promote global knowledge exchange.

Abbreviation: AILD, autoimmune liver diseases.

## CONCLUSIONS

Autoimmune liver diseases (AILDs), such as primary biliary cholangitis (PBC) and autoimmune hepatitis (AIH), continue to be underdiagnosed and insufficiently characterized in Latin America. Despite growing global awareness of these conditions in recent years, the region still faces critical challenges in diagnostic capacity, access to treatments, and research infrastructure. Healthcare disparities, unequal distribution of resources, and a lack of high-quality local clinical and epidemiological data hinder the development of evidence-based strategies tailored to the specific needs of the region. Strengthening regional collaboration and supporting multicenter research initiatives are key to bridging these gaps and improving the clinical management of these diseases. At the same time, public policies ensuring equitable access to treatments and interventions, such as liver transplantation, are vital to addressing disparities in patient outcomes. Engagement from the pharmaceutical industry, through responsible pricing strategies and philanthropic efforts, may also contribute to expanding access to essential therapies in resource-limited settings.

Regarding PBC, limited access to diagnostic tools, such as AMA testing and liver biopsy, often leads to delayed or missed diagnoses. A distinct immunological phenotype previously reported in Latin American populations, characterized by a higher prevalence of AMA-negative cases and a histological profile with prominent inflammatory activity, may add further complexity to the diagnostic process and warrants further research.

Many patients are diagnosed at advanced stages, diminishing the potential benefits of early intervention and contributing to poorer outcomes. To address this, urgent efforts are needed to raise awareness among primary care and specialist physicians, streamline referral pathways, and ensure equitable access to both first-line therapies, such as ursodeoxycholic acid, and second-line options, like fibrates. Tackling these challenges requires targeted strategies to enhance disease recognition, simplify referral processes, and guarantee access to effective treatments across all patient demographics.

Similarly, AIH in Latin America presents unique challenges. Regional studies consistently demonstrate a higher proportion of patients presenting with advanced disease, including acute severe hepatitis, fulminant hepatic failure, and established cirrhosis at the time of diagnosis. Although most patients receive standard immunosuppressive therapy, treatment response may be suboptimal in a subset of individuals, with early progression to portal hypertension despite adherence to recommended regimens. These observations suggest the involvement of underlying genetic, environmental, and socioeconomic factors influencing disease phenotype and therapeutic outcomes. Moreover, limited access to liver transplantation—a life-saving therapy for patients with acute liver failure or end-stage disease- further exacerbates inequities in long-term prognosis.

Improving outcomes for patients with AILDs in Latin America requires a comprehensive, multifaceted strategy that is adapted to the region’s specific needs and disparities.

In conclusion, both PBC and AIH remain underdiagnosed, undertreated, and under-researched in Latin America, despite their significant contribution to chronic liver disease and the liver transplantation burden. Large epidemiological studies using standardized methodologies are needed to better understand the prevalence and management of AILD in Latin America and to inform strategies for improvement. Two important studies are currently underway. The first one is a retrospective, multicenter study sponsored by the ALEH in collaboration with several national scientific societies, aimed at evaluating the prevalence, management, and outcomes of AILD in the region. The second is a prospective study conducted at Hospital Italiano in Argentina, with the support of ALEH. The results of both initiatives are highly anticipated. Various initiatives are beginning to address the long-standing gaps by generating high-quality data on the epidemiology, clinical management, and outcomes of AILD in the region. Sustained support for these efforts—alongside the development of national disease registries and the strengthening of collaborative research networks—will be essential not only for the formulation of regionally adapted clinical practice guidelines but also for informing public health policies that prioritize timely diagnosis, equitable access to therapies, and optimized use of healthcare resources. These data-driven strategies have the potential to reduce disparities in care, improve patient outcomes, and ultimately elevate the standard of hepatology practice across Latin America.

Only through coordinated and sustained efforts that integrate clinical care, research, and policy actions will it be possible to close existing gaps and provide equitable, high-quality care for patients across the region. Latin America has the opportunity, and the responsibility, to lead region-specific initiatives that contribute meaningfully to the global understanding and management of autoimmune liver diseases.
